# High production of CH_4_ and H_2_ by reducing PET waste water using a non-diaphragm-based electrochemical method

**DOI:** 10.1038/srep20512

**Published:** 2016-02-04

**Authors:** Nam-Gyu Kim, Kwang-Jin Yim, Chan-Soo Kim, Dong-Keun Song, Kikuo Okuyama, Min-ho Han, Young-hoo Kim, Sung-Eun Lee, Tae-Oh Kim

**Affiliations:** 1 Department of Environmental Engineering, Kumoh National Institute of Technology, Daehak-ro 61, Gumi, Gyeongbuk 730-701, Republic of Korea; 2 Department of Eco-Machinery Systems, Environmental and Energy Systems Research Division, Korea Institute of Machinery and Materials, 156 Gajeongbuk-ro, Yuseong, Daejeon 305-343, Republic of Korea; 3 Marine Energy Convergence & Integration Laboratory, Jeju Global Research Center, Korea Institute of Energy Research, Republic of Korea; 4 Department of Chemical Engineering, Graduate School of Engineering, Hiroshima University, 1-4-1 Kagamiyama, Higashi Hiroshima 739-8527, Japan; 5 School of Applied Biosciences, Kyungpook National University, Daegu 702-701, Republic of Korea

## Abstract

In recent years, the worldwide use of polyethylene terephthalate (PET) has increased exponentially. PET wastewater contains ethylene glycol (EG) and terephthalic acid (TPA). In this study, we present a unique method for producing combustible gases like CH_4_ and H_2_ from PET wastewater by electrochemical reaction of EG and TPA. The non-diaphragm-based electrochemical (NDE) method was used to treat PET wastewater. The electrochemical removal of EG and TPA from PET wastewater was examined and the optimal conditions for their reduction to CH_4_ and H_2_ were determined. Using the proposed system, 99.9% of the EG and TPA present in the PET wastewater samples were degraded to produce CH_4_ and H_2_, at applied voltages lower than 5 V. The highest Faradaic efficiency achieved for EG and TPA reduction was 62.2% (CH_4_, 25.6%; H_2_, 36.6%), at an applied voltage of 0.8 V. Remarkably, CH_4_ was produced from EG decomposition and H_2_ from TPA decomposition. To the best of our knowledge, this is the first reported instance of CH_4_ and H_2_ production from EG and TPA, respectively. The electrochemical reductive treatment will be an important discovery for reducing water contamination and replacing fossil fuels with respect to generating green energy.

The production of synthetic fibers of polyethylene terephthalate (PET) has increased exponentially in recent years^1^,^2^,^3^,^4^,^5^,^6^. PET can be synthesized directly by polymerization of ethylene glycol (EG) and terephthalic acid (TPA), or from dimethyl terephthalate (DMT) by what is known as the DMT method^7^,^8^,^9^,^10^.

Some of the raw materials used for PET production, such as EG and TPA, are extracted from natural gases and crude oils, and are very expensive. Unused amounts of these compounds are, therefore, collected and recycled following PET production, using methanolysis, ethanolysis, and glycolysis^3^,^11^,^12^,^13^,^14^. Although the aforesaid recycling processes allow for the recovery of most of the EG and TPA, small quantities (<0.2%) of these compounds remain in solution and are discharged into the environment. This effluent is known as PET polymerization wastewater^15^,^16^,^17^.

To remove EG and TPA from PET wastewater, PET manufacturers employ activated carbon filters or advanced water-treatment methods such as anaerobic digestion and ozone treatment. However, these methods are expensive and inefficient^17^,^18^,^19^,^20^. Recently, it is reported that electrochemical treatments can efficiently remove EG or TPA through a redox reaction driven by a direct current power source^25^,^26^,^27^,^28^,^29^. These results also reported that electrochemical treatment can produce combustible gases for use as fuels.

Until now, various technologies have been applied to remove EG and TPA from PET wastewaters^30^,^31^,^32^,^33^. However, no one has considered EG and TPA as energy sources. Electrochemical reduction of CO_2_ can produce ethylene (C_2_H_4_) and EG, which in turn can be converted to methane (CH_4_) and ethane (C_2_H_6_)^34^,^35^,^36^. These findings suggest that EG and TPA from PET wastewater could be converted into energy sources because of their structural characteristics, as well as their carbon and hydrogen contents. Therefore, the research into methods to transform the wastewater contents into energy sources should necessarily be combined with an efficient method for their removal.

In this study, we present a unique method for producing combustible gases such as CH_4_ and H_2_ from PET wastewater by electrochemical reaction of EG and TPA. Furthermore, to the best of our knowledge, this is the first reported study on the cost reduction of PET wastewater treatment. The electrochemical reductive treatment is a potentially important method for decreasing water contamination and for replacing fossil fuels with respect to generating green energy.

This study examines the electrochemical removal of EG and TPA from PET wastewater and determines the optimal conditions for their reduction to CH_4_ and H_2_, to develop a method for producing fuels from waste via wastewater treatment.

## Results

### Current density as a function of the applied voltage and the electrolyte

Electrochemical analysis was conducted to elucidate the mechanisms of EG and TPA reductions in PET wastewater generating CH_4_ and H_2_. To analyze electrochemical power, linear-sweep voltammetry (LSV) and cyclic voltammetry (CV) experiments were conducted using an electrochemical analyzer (PAR VersaSTAT3, AMETEK).

Figure S1 shows the results of LSV with three 50 wt% methanol solutions: the first with 0.2 M KOH, the second with 0.2% EG and TPA content, and the third containing both. Electrical current was not generated in the absence of KOH, indicating that KOH is an essential ingredient for the electrochemical reduction of EG and TPA. KOH generates OH radicals, a useful property for application in electrochemical reactions^44^,^45^, which play an important role in reducing EG and TPA to CH_4_ and H_2_. When KOH has a lower bonding energy than H_2_O, KOH is dissociated into K^+^ ions and OH radicals at low electric potential, and its electric currents flow. Produced K^+^ ions induce to decompose H_2_O and generating H^+^ ions and OH radicals. The excessive OH radicals generated by the above reaction possess higher reaction rates than proton and are strong oxidizing agents for dissolved organic matter (DOM). These OH radicals attack benzene rings of TPA and EG. The produced H_2_O in the process of EG decomposition transforms into OH radicals and recycles the TPA and EG decomposition^52^,^53^,^54^,^55^. This KOH electrolyte was mixed with methanol because the methanolysis has generally been used to produce biogases^17^,^18^,^41^,^42^,^43^. However, KOH is a strongly alkaline substance requiring an appropriate pH for reaction in the general diaphragm electrochemical method^46^,^47^,^48^. Our NDE method system was unicellular, pH-independent, and showed highly efficient generation and flow of electrons.

Electrical current was observed both in presence and absence of EG and TPA in the NDE method cell. Especially, current density abruptly increased from a potential of −0.5 V onwards. Therefore, electric power greater than −0.5 V was acceptable for degrading EG and TPA to produce CH_4_ and H_2_, indicating that increasing potential causes greater degradation of EG and TPA.

**Figure 1:**
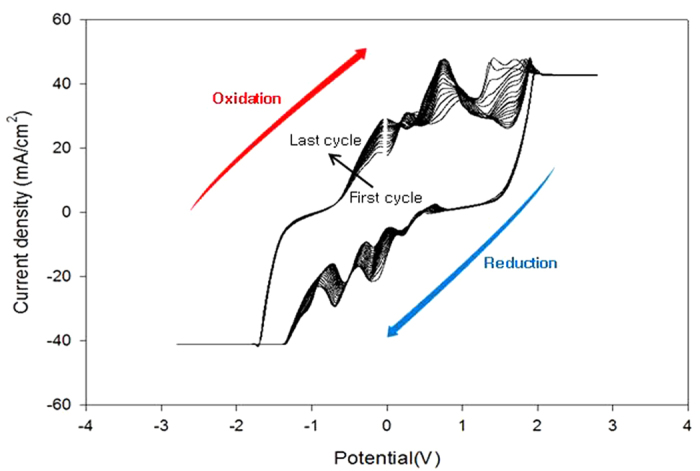
Cyclic voltammogram (Cu electrode) with varying potentials and impedance (IMP) values, using the NDE method on a 0.2 M KOH solution containing 0.2% EG/TPA and 50 wt% methanol (scan rate 50 mVs^−1^, 25 °C, frequency 10,000 Hz, amplitude 500 mV, NDE method).

Current densities were measured for voltages ranging from −3 to 3 V applied to a 0.2 M KOH solution in methanol (50 wt%) containing 0.2% EG/TPA (Fig. 1). High current densities were observed at relatively low applied voltages, indicating that current density increased when the applied voltage exceeded 0.2 V. After the voltages were added, small reduction peaks were generated at the 0.2 V. This meant that the reduction was initiated at 0.2 V, and the reduction occurred preferentially compared to the oxidation reaction, at 0.5 V. The highest reduction peak was obtained at 0.8 V, with increasing numbers of CV cycles. This showed that the reduction of EG/TPA presumably happened at 0.8 V, and many products (CH_4_ and H_2_) were produced at low added voltages. To evaluate this reaction pattern, EG and TPA degradations were analyzed by applying voltages of various magnitudes, for different durations.

### Comparison of CH_4_ and H_2_ production rates

**Figure 2:**
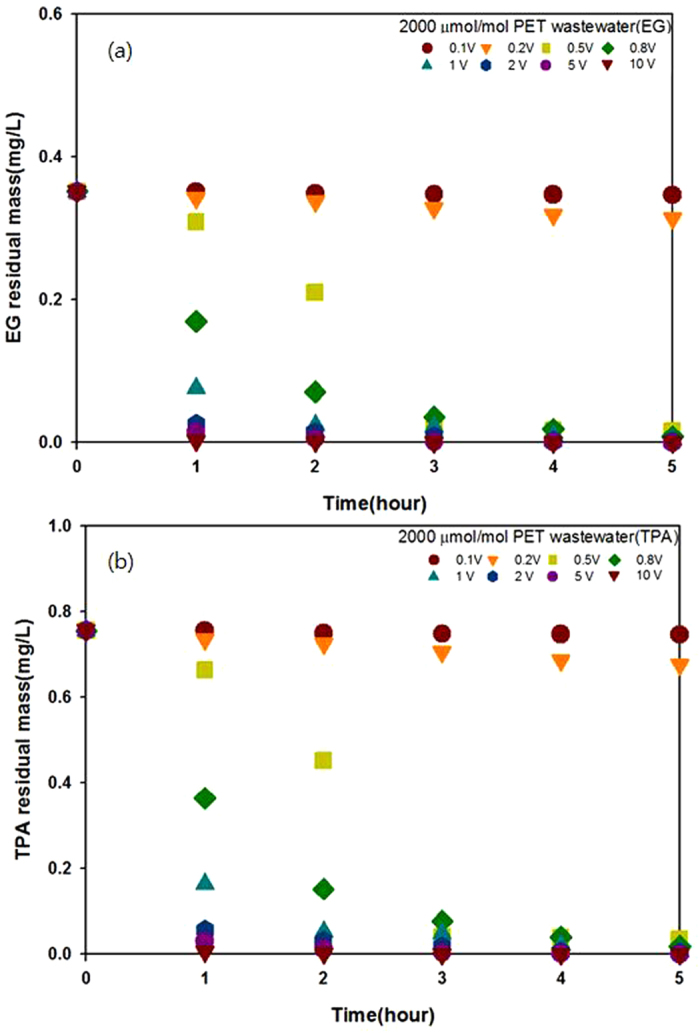
Residual masses of (**a**) EG and (**b**) TPA as a function of time for different applied voltages using the NDE method (0.2 M KOH electrolyte, 50 wt% methanol, 25 °C, Cu working electrode, Pt counter electrode, 1 atm).

Figure 2 shows the hourly results of residual mass obtained from a 0.2% EG solution and a 0.2% TPA solution. The solutions were incubated for 5 h, with applied voltages in the 0–10 V range used to determine their degradation patterns. At applied voltages below 0.2 V, EG and TPA did not degrade sufficiently. However, sufficient degradation was observed at voltages as low as 0.5 V. A voltage of 2 V applied for 1 h degraded EG and TPA by as much as 90%, while an applied voltage of 5 V achieved 99% degradation in the same period. These results are consistent with the CV results (Fig. 1), which indicated the current required to degrade EG and TPA.

From these results, it can be concluded that a 0.2% EG and a 0.2% TPA solution can be efficiently degraded with an applied voltage of 5 V.

**Figure 3:**
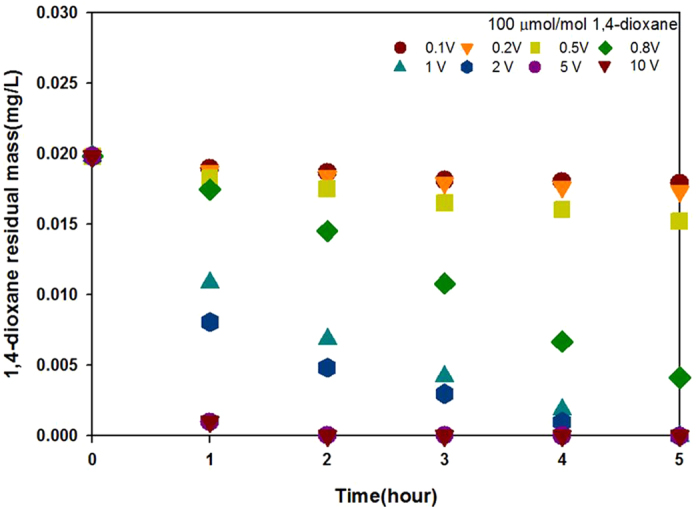
Residual mass of 1,4-dioxane as a function of time for different applied voltages using the NDE method (conditions as described in Fig. 2).

CO_2_ and 1,4-dioxane are intermediates and byproducts in the polymerization reaction involving EG and TPA. GC-MSD analyses of the decomposition products confirmed these intermediates and other byproducts (Fig. S2, Supplementary Information). 1,4-dioxane was generated with a fractional rate (~100 μmol/mol) in the polymerization of a 0.2% solution of EG and TPA sampled from PET wastewater, consistent with the industrial process of PET production. As is common, 1,4 dioxane content has been used as a marker for the efficiency of PET wastewater treatments in this study. The residual mass was determined for a variety of applied voltages over 5 h (Fig. 3). The degradation pattern observed for 1,4-dioxane was similar to that for EG/TPA. Applied voltages greater than 0.5 V were more effective than the lower voltages. Thus, the efficiency of 1,4-dioxane degradation increases with increasing voltage. This is the first demonstration of an NDE method proving highly efficient at 1,4-dioxane removal. This study demonstrated that applied voltages in the 0–10 V range can degrade EG, TPA, and 1,4-dioxane.

We next conducted a series of GC experiments in order to determine the degradation products of EG, TPA, and 1,4-dioxane. Since EG and TPA are mainly composed of carbon and hydrogen, the main reduction products were CH_4_ and H_2_.

**Figure 4:**
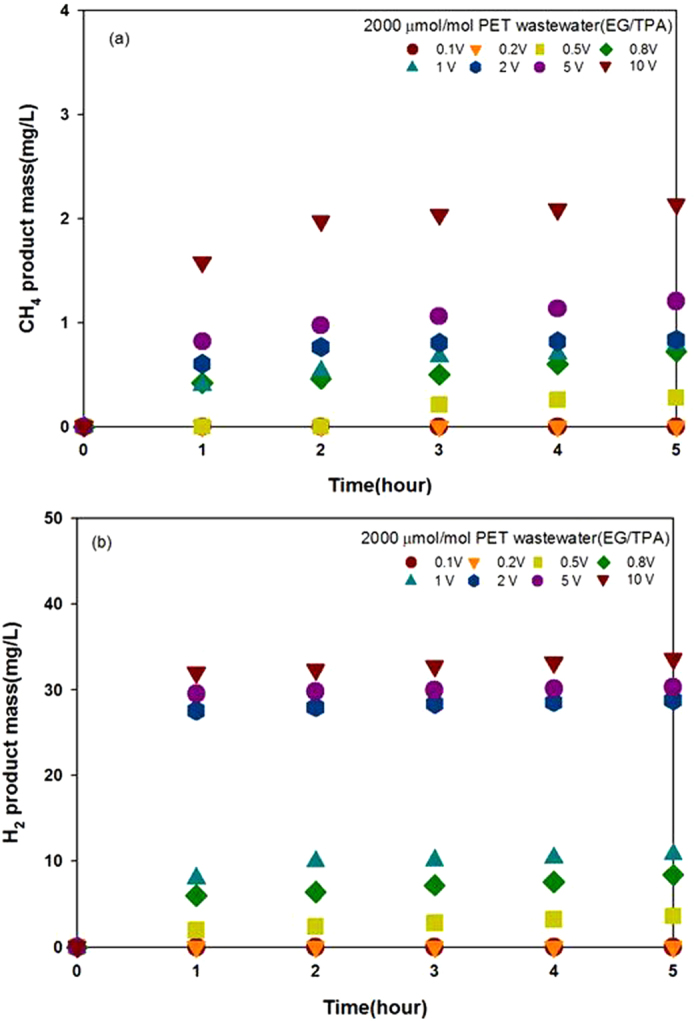
CH_4_ and (**b**) H_2_ produced as a function of time for various applied voltages (conditions as described in Fig. 2).

The amounts of CH_4_ and H_2_ produced (shown in Fig. 4a,b, respectively) increased with increasing applied voltage. CH_4_ production was largely unchanged over the voltage range 0.5–5 V. H_2_ production was fifteen times greater than CH_4_ production at applied voltages ≤2 V. In the absence of an applied voltage, CO_2_ was the only product detected from the solution (Fig. S3, Supplementary Information).

**Figure 5:**
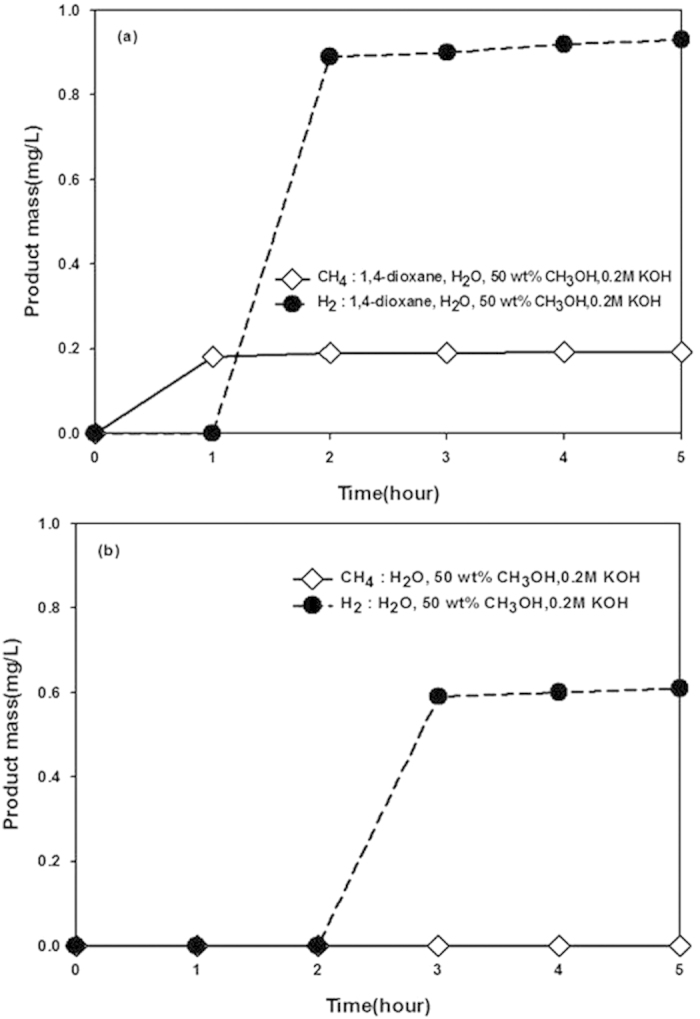
Masses of CH_4_ and H_2_ produced as a function of time under 0.8 V applied voltage. Reaction mixture (**a**) contains 1,4-dioxane, while (**b**) does not (conditions as described in Fig. 2).

These results represent an important step forward in PET wastewater treatment, not only for removing a carcinogen from both CH_4_ and H_2_ were produced through EG and TPA degradation in the NDE method. H_2_O and CH_3_OH, used as electrolytes, were also expected to contribute to CH_4_ and H_2_ production. For further investigation, a blank experiment was run in the absence of EG and TPA (Fig. 5). In the reaction mixture containing 100 μmol/mol 1,4-dioxane (Fig), both CH_4_ and H_2_ were produced. However, in the reaction mixture containing only H_2_O, 50 wt% CH_3_OH, and KOH, only H_2_ was produced (albeit in a smaller amount) and CH_4_ was not (Fig. 5b), by the electrochemical conversion of H_2_O^43^,^49^,^50^,^51^. These results demonstrated that 1,4-dioxane participates in the production of both CH_4_ and H_2_. The concentration of 1,4-dioxane gradually decreased over time (Fig. 3), providing further evidence for its involvement in CH_4_ and H_2_ production.

Further experiments were performed to understand the role of CO_2_ in the production of CH_4_ and H_2_. In Fig S3, CO_2_ concentration was constantly maintained after 1 hr. In Fig S4, CH_4_ was not produced. Therefore, CO_2_ cannot be considered as a carbon source in the reaction, as it did not play any role in CH_4_ production. In Fig S4, the increasing voltages were related to electrolysis of water, increasing the H_2_ concentration. Therefore, CO_2_ is an intermediate which is not related to the CH_4_ and H_2_ generation.

### Faradaic efficiency

**Figure 6:**
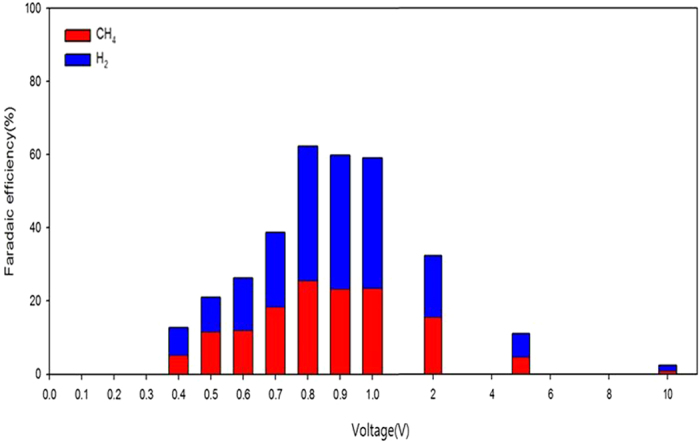
Faradaic efficiencies of CH_4_ and H_2_ productions versus applied voltage (conditions as described in Fig. 2, except that the reaction mixture contains 0.2% EG/TPA).

The Faradaic efficiencies of CH_4_ and H_2_ production were calculated for a range of voltages (0–10 V) applied to a reaction mixture containing 0.2 M KOH, 0.2% EG/TPA, and 50 wt% CH_3_OH (Fig. 6). Current density and Faradaic efficiency were both enhanced by increasing the applied voltage from 0.2 to 0.8 V. The highest Faradaic efficiency, 62.2% (CH_4_, 25.6%; H_2_, 36.6%), was achieved at 0.8 V. This result is consistent with those of LSV and CV (Fig. S1, Fig. 1), with the current density and Faradaic efficiency increasing with increase in applied voltage up to 0.8 V. However, Faradaic efficiency decreased at voltages above 0.8 V, decreasing to less than 10% at 5 V. These results indicated that the removal of EG and TPA, and the consequent production of CH_4_ and H_2_ are best achieved in this electrochemical system at an applied voltage of 0.8 V. Therefore, applied voltages <1 V are most suited to CH_4_ and H_2_ production from PET wastewater.

**Figure 7:**
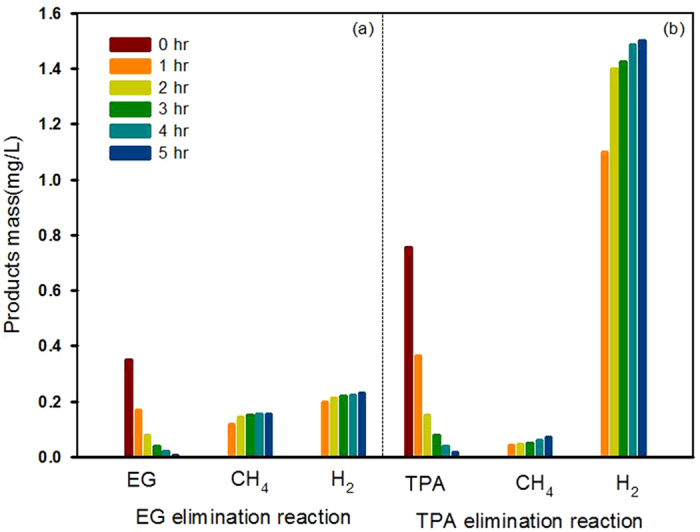
Production of CH_4_ and H_2_ with elimination of (**a**) EG and (**b**) TPA as a function of time (reaction conditions as described in Fig. 2).

A series of experiments was conducted to determine the mechanisms of CH_4_ and H_2_ production from EG or TPA. It was determined that CH_4_ is the predominant product of the reaction mixture that contained only EG (Fig. 7a), while H_2_ production was dramatically higher in the reaction mixture containing only TPA (Fig. 7b). The reaction mixture containing both EG and TPA generated three times more CH_4_ and H_2_ than the samples containing either EG or TPA (Fig. S6, Supplementary Information). From these results, we concluded that CH_4_ production is primarily affected by the EG content, while TPA content primarily affects H_2_ production. The absolute amounts of CH_4_ and H_2_ produced are a result of TPA degradation.

**Figure 8:**
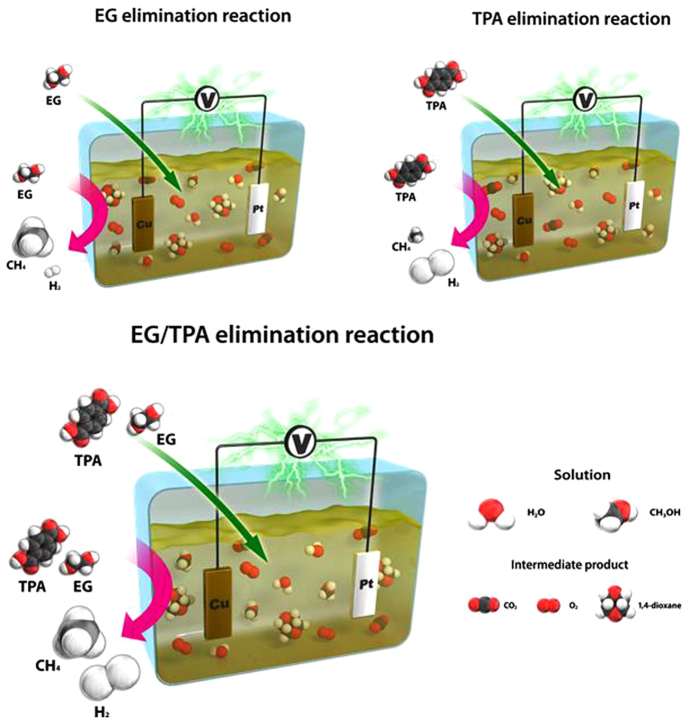
Schematics for the electrochemical reduction of EG/TPA (conditions as described in Fig. 2).

We performed 30 day experiments to prove physiochemical stabilities of Cu and Pt electrodes in electrolytes through reactions. Measurements were taken twice a day for one month. Within these days, abrasion and leaching of Cu and Pt electrodes were not found. Faradaic efficiency was presented using the results obtained from these experiments (Fig. S5). With the batch experiment, Faradaic efficiency of CH_4_ and H_2_ production decreased for the first 5 days because the supplied and consumed reactants were limited. Afterwards, the Faradaic efficiency was constantly maintained throughout the 30 days. These results indicate the stability of Cu and Pt electrodes in the electrolytes through the reaction. A schematic explaining the electrochemical reactions of EG/TPA and the reactions that generate CH_4_ and H_2_ from EG and TPA, respectively, are shown in Fig. 8.

### Reaction mechanism

We have demonstrated that CH_4_ and H_2_ are produced by reducing PET wastewater via NDE. During this process, applied voltages generated electronic and protonic currents, which initiated EG and TPA reduction within the mixture. Likely reaction mechanisms are proposed below.


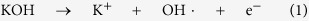



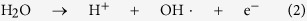



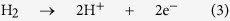



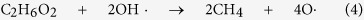



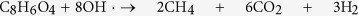



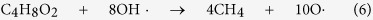


From equations (1) and (2), OH radicals generated from KOH and H_2_O electrolyses were strong oxidizing agents to degrade EG, TPA and 1,4-dioxane. From Equation (4), CH_4_ was produced after OH radicals reacted with EG. It is concurrent to the results of Fig. 7 showing that production of methane is primarily related to EG. At Equation (5), TPA generated methane, H_2_, and CO_2_ after reactions with 8 OH radicals, and it is considered to preferentially produce H_2_ rather than CH_4_ gas. The intermediate CO_2_ gas is equivalent to the analytical results shown in Fig. S2. At Equation (6), 1,4-dioxane is reduced to methane after reactions with OH radicals and removed.

## Discussion

In this study, an NDE method was developed to produce high-value fuels from EG and TPA in PET wastewater by applying relatively low voltages. Nearly 99.9% of the EG and TPA present in PET wastewater samples were removed with an applied voltage of 5 V. The highest Faradaic efficiency for EG and TPA reduction was 62.2% (CH_4_, 25.6%; H_2_, 36.6%), achieved using an applied voltage of 0.8 V. Based on our findings, we have proposed reaction mechanisms describing CH_4_ and H_2_ production, suggesting that EG produces only CH_4_, while TPA produces both CH_4_ and H_2_. Overall, valueless and environmentally hazardous PET wastewater was easily treated, using a simple electrochemical method, to produce high-value fuels (CH_4_ and H_2_) from EG and TPA. This report represents a proof of concept, and further pilot-scale studies will be required before the technology can be marketed.

## Methods

### Electrochemical reduction

We have previously reported the development of a highly efficient, high-current-density non-diaphragm-based electrochemical (NDE) method to achieve electrochemical reduction^35^. The same method, involving the use of acrylic materials (60 mm × 45 mm × 95 mm), was applied to the removal of EG and TPA from PET wastewater. There are many reports, that hydrocarbons such as CH_4_ and C_2_H_4_, are easily produced using the Cu cathode. Cu is also cheaper than other metal electrodes, which is why the cu electrode was used as working electrodes to produce CH_4_^56^,^57^,^58^. Insoluble Pt and Cu plates were used as anode and cathode, respectively. The plates had same dimensions (40 mm × 40 mm × 1 mm), were positioned at the same height, with 10-mm separation. Ag/AgCl electrolyte surrounded the electrodes. The entire electrochemical system was incorporated into an electrochemical analyzer (PAR VersaSTAT3, AMETEK). Experiments were conducted for 5 h on EG (99.9%, Sigma Aldrich) and TPA (99.9%, Sigma Aldrich) using 0.2 M KOH (Extra pure, Sigma Aldrich) electrolyte solutions containing 50 wt% CH_3_OH (99.999%, Sigma Aldrich). Voltages were applied over the range 0–10 V and the generated currents were measured.

### CH_4_ and H_2_ production under various conditions

The gas-phase products were collected from an outlet placed near the top of the NDE apparatus. The compositions of the collected samples were determined by gas chromatography (GC) using a flame ionization detector (FID, Agilent HP6970) for CH_4_, or a thermal conductivity detector (TCD, PerkinElmer CALUS580) for H_2_. Various intermediates and by-products, including 1,4-dioxane, were analyzed using a mass-selective detector (MSD, Agilent HP6970). The results of these analyses were used to calculate the Faradaic efficiencies and thus to determine the fate of EG and TPA.

## Additional Information

**How to cite this article**: Kim, N.-G. *et al.* High production of CH_4_ and H_2_ by reducing PET waste water using a non-diaphragm-based electrochemical method. *Sci. Rep.* **6**, 20512; doi: 10.1038/srep20512 (2016).

## Supplementary Material

Supplementary Information
