# Supplementary material for: High production of CH4 and H2 by reducing PET waste water using a non-diaphragm-based electrochemical method
Source: Sci Rep. 2016 Feb 4;6:20512. doi: 10.1038/srep20512 (PMC4740802; doi:10.1038/srep20512)
Supplement: Supplementary Information [file srep20512-s1.doc]

**Supporting Information**

**High production of CH_4_ and H_2_ by reducing PET waste water using a non-diaphragm-based electrochemical method**

Nam-Gyu Kim,‡^1^ Kwang-Jin Yim,‡^1^ Chan-Soo Kim,‡^3^ Dong-Keun Song,^2^ Kikuo Okuyama,^4^ Min-ho Han^1^, Young-hoo Kim^1^, Sung-Eun Lee*^5^ and Tae-Oh Kim*^1^

*^1^*Department of Environmental Engineering, Kumoh National Institute of Technology, Daehak-ro 61, Gumi, Gyeongbuk 730-701, Republic of Korea. tokim@kumohac.kr; Fax: +82-54-478-7641; Tel: +82-54-478-7634.

*^2^*Department of Eco-Machinery Systems, Environmental and Energy Systems Research Division, Korea Institute of Machinery and Materials, 156 Gajeongbuk-ro, Yuseong, Daejeon 305–343, Republic of Korea

*^3^*Marine Energy Convergence & Integration Laboratory, Jeju Global Research Center, Korea Institute of Energy Research, Republic of Korea

*^4^*Department of Chemical Engineering, Graduate School of Engineering, Hiroshima University, 1-4-1 Kagamiyama, Higashi Hiroshima 739-8527, Japan

*^5^*School of Applied Biosciences, Kyungpook National University, Daegu 702–701, Republic of Korea. E-mail: selpest@knu.ac.kr; Fax: +82-53-953-7233; Tel: +82-53-950-7768

**
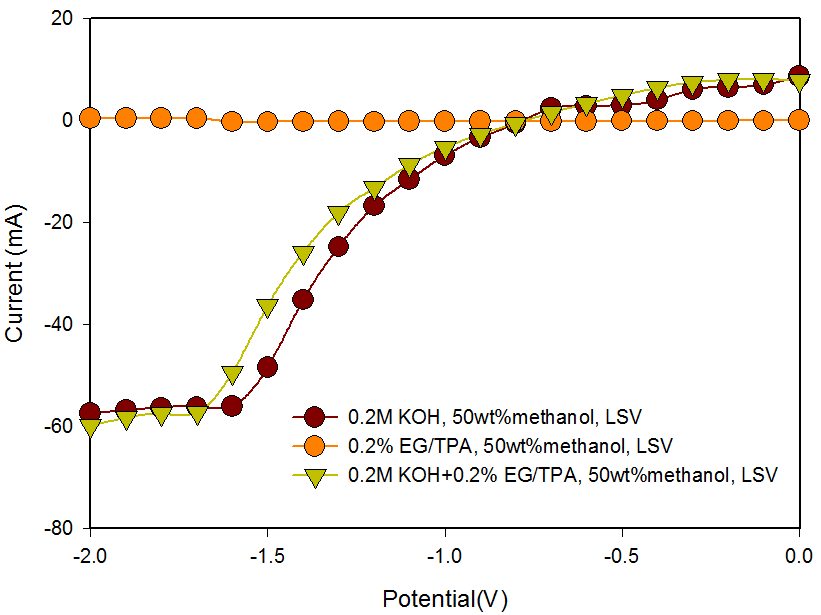
**

**Figure S1│ Linear sweep voltammetry (Cu electrode), with different potentials and impedance (IMP) values, of three solutions (voltammetry settings as described in Fig. 1).**

**
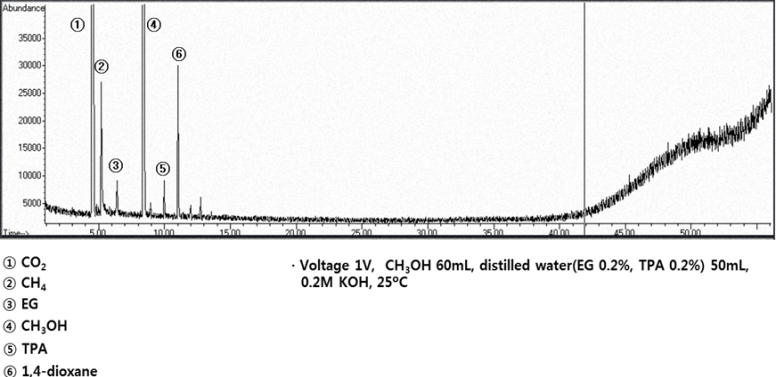
**

**
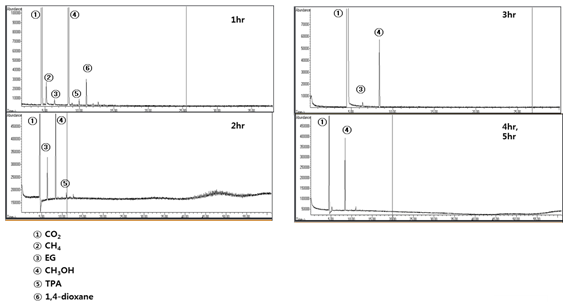
**

**Figure S2│ GC-MSD analysis of products and components of the 0.2 % EG/TPA solution.**

**
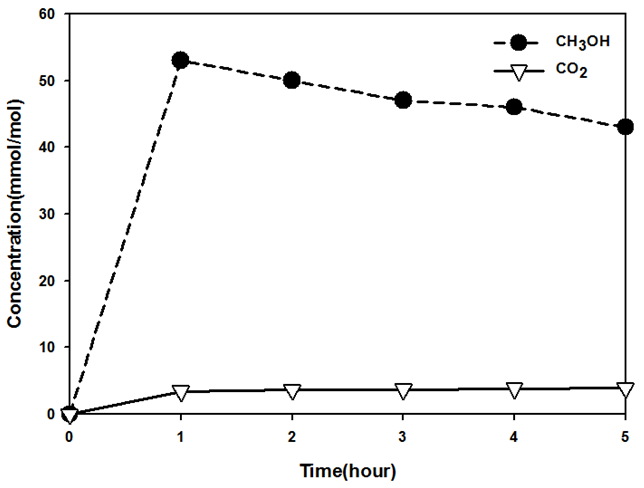
**

**Figure S3│ CO_2_ and CH_3_OH contents of the 0.2% EG/TPA solution as a function of time (conditions as described in Figure. 2)**

**
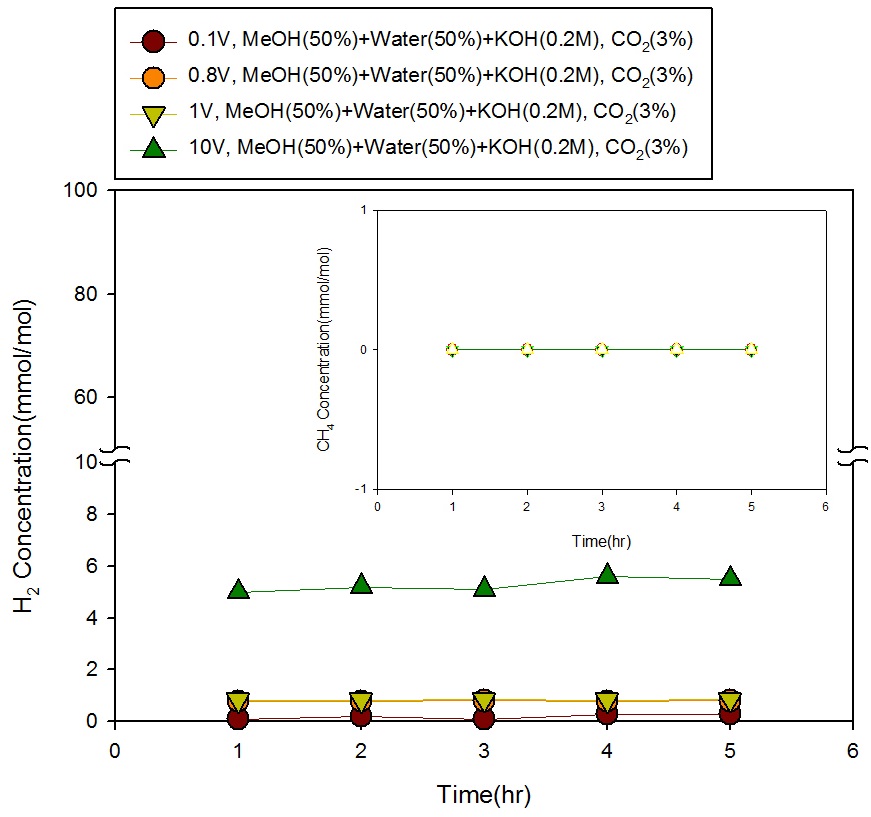
**

**Figure S4│ CH_4_ and H_2_ concentrations of solutions containing CO_2_, water, 0.2 M KOH, and 50 wt% CH_3_OH, as a function of time, for various applied voltages (conditions as described in Fig. 2)**

**
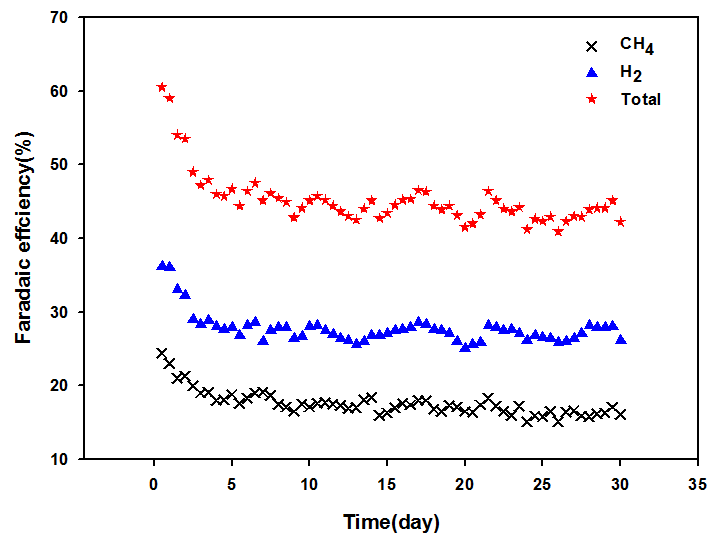
**

**Figure S5│ Faradaic efficiencies of CH_4_ and H_2_ production from EG/TPA solution for one month (conditions as described in Fig. 2)**

**
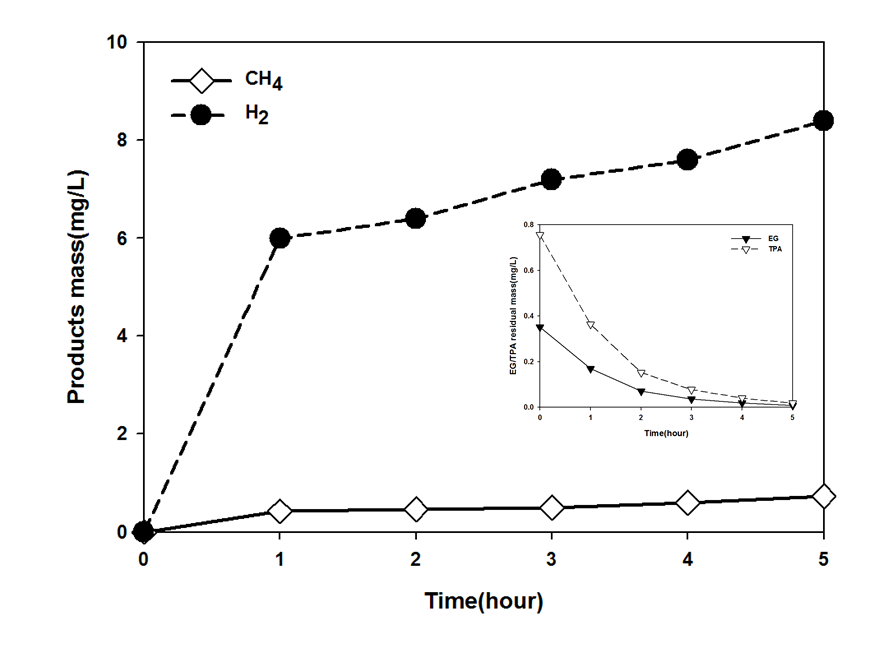
**

**Figure S6│ Production of CH_4_ and H_2_, and elimination of EG and TPA, as a function of time, (conditions as described in Fig. 2)**

**REFERENCES**

1. Li, X.-K., Lu ,H., Guo, W.-Z. Cao, G.-P., Liu, H.-L., Shi, Y.-H. Reaction kinetics and mechanism of catalyzed hydrolysis of waste PET using solid acid catalyst in supercritical CO_2_. *AIChE J.* **1**, 200–214 (2015).
2. Hu, L.-C., Oku, A., Yamada, E., Tomari, K. Alkali-decomposition of poly (ethylene terephthalate) in mixed media of nonaqueous alcohol and ether. Study on recycling of poly (ethylene terephthalate). *Polym.* J. **29**, 708–712 (1997).
3. Aguado, A., Martínez, L., Becerra, L., Arieta-Araunabeña, M., Arnaiz, S., Asueta , A., Robertson, I. Chemical depolymerisation of PET complex waste: hydrolysis vs. glycolysis. *J. Material Cycles Waste Manage.* **16**, 201–210 (2014).
4. Liu, Y., Wang, M., Pan, Z. Chemical recycling of waste poly (ethylene terephthalate) fibers into azo disperse dyestuffs. *J. Supercrit. Fluids*. **62**, 226–231 (2012).
5. Sinha, V., Patel, M. R., Patel, J. V. PET waste management by chemical recycling: a review. *J. Polym. Environ*. **18 (1)**, 8–25 (2010).
6. López-Fonseca, R., Duque-Ingunza, I., de Rivas, B., Arnaiz, S., Gutiérrez-Ortiz, J. I. Chemical recycling of post-consumer PET wastes by glycolysis in the presence of metal salts. *Polym. Degrad. Stab.* **95**, 1022–1028 (2010).
7. Oku, A., Hu, L. C., Yamada, E. Alkali Decomposition of Poly(ethylene terephthalate) with Sodium Hydroxide in Nonaqueous Ethylene Glycol: A Study on Recycling of Terephthalic Acid and Ethylene Glycol. *J. Appl. Polym. Sci.* **63**, 595–601 (1997).
8. Vaidya, U. R., Nadkarni, V. M. Polyester polyols from glycolyzed PET waste: effect of glycol type on kinetics of polyesterification. *J. Appl. Polym. Sci.* **38**, 1179–1190 (1989).
9. Siddiqui, M. N., Achilias, D. S., Redhwi, H. H., Bikiaris, D. N., Katsogiannis, K.-A. G., Karayannidis, G. P. Chemical recycling of polycarbonate based wastes using alkaline hydrolysis under microwave irradiation. *Macromol. Mater. Eng.* **295**, 575–584 (2010).
10. Imran, M., Kim B.-K., Han, M., Cho B. G., Kim, D. H. Kinetics of catalytic glycolysis of PET wastes with sodium carbonate. *Polym. Degrad. Stab.* **95**, 1686–1693 (2010).
11. Chen, J.Y., Ou, C.F., Hu, Y.C., Lin, C.C. Depolymerization of poly(ethylene terephthalate) resin under pressure. *J. Appl. Polym. Sci.* **42**, 1501–1507 (1991).
12. Sako, T., Sugeta, T., Otake, K., Nakazawa, N., Sato, M., Namiki, K., Tsugumi, M. Depolymerization of polyethylene terephthalate to monomers with supercritical methanol. *J. Chem. Eng. Jpn.* **30 (2)**, 342–346 (1997).
13. Wang, Q., Geng, Y., Lu, X., Zhang, S. Chemical recycling of unsaturated polyester resin and its composites via selective cleavage of the ester bond. *ACS Sustainable Chem. Eng.* **3**, 340−348 (2015).
14. Paszun, D., Spychaj, T. Chemical recycling of poly (ethylene terephthalate). *Ind. Eng. Chem. Res.*, **36**, 1373 (1997).
15. Viana, M. E., Riul, A., Carvalho, G. M., Rubira, A. F., Muniz, E. C. Chemical recycling of PET by catalyzed glycolysis: Kinetics of the heterogeneous reaction. *Chem. Eng. J.* **173**, 210–219 (2011).
16. Shah, R. V., Borude , V. S., Shukla, S. R. Recycling of PET waste using 3‐amino‐1‐propanol by conventional or microwave irradiation and synthesis of bis‐oxazin there from. *J. Appl. Polym. Sci.* **127**, 323–328 (2013).
17. Yoshioka, T., Sato, T., Okuwaki, A. Hydrolysis of waste PET by sulfuric acid at 150°C for a chemical recycling. *J. Appl. Polym. Sci.* **52**, **9**, 1353–1355 (1994).
18. Hamelers, H. V., Heijne, A. T., Sleutels, T. H., Jeremiasse, A. W., Strik, D. P., Buisman, C. Hydrogen gas production in a microbial electrolysis cell by electrohydrogenesis. *Appl. Microbial. Biotechnol.* **85 (6)**, 1673–1685 (2010).
19. Heng, S., Yeung, K. L., Djafer, M., Schrotter, J.-C. A novel membrane reactor for ozone water treatment. *J. Membr. Sci.* **289**, 67–75 (2007).
20. Heng, S., Yeung, K. L., Julbe, A., Ayral, A., Schrotter, J.-C. Preparation of composite zeolite membrane separator/contactor for ozone water treatment. *Microporous Mesoporous Mater.* **115**, 137–146 (2008).
21. Kim, H. J., Choi, S. M., Green, S., Tompsett, G. A., Lee, S. H., Huber, G. W., Kim, W. B. Highly active and stable PtRuSn/C catalyst for electrooxidations of ethylene glycol and glycerol. *Appl. Catal. B: Environ.* **101**, 366–375 (2011).
22. Lv, S., Wang, C., Yang, X. Hierarchical semiconductor oxide photocatalyst: a case of the SnO_2_ microflower. *Micro Nano Lett.* **8**, **(5)**, 234–237 (2013).
23. Zhang, Z. J., Cui, P., Chen, X. Y. Structure and Capacitive Performance of Porous Carbons Derived from Terephthalic Acid–Zinc Complex via a Template Carbonization Process. *Ind. Eng. Chem. Res.* **52**, 16211−16219 (2013).
24. Hu, S., Tian, R., Dong, Y., Yang, J., Liu, J., Chang, Q. Modulation and effects of surface groups on photoluminescence and photocatalytic activity of carbon dots. *Nanoscale*, **5**, 11665–11671 (2013).
25. Sako, T., Sugeta, T., Otake, K., Takebayashi, Y., Kamizawa, C., Tsugumi, M., Hongo, M., Ronbunshu, K. Depolymerization of Polyethylene Terephthalate to Monomers with Supercritical Methanol *J. JCEJ.* **55**, **11**, 685–690 (1998).
26. Anand, M. V., Srivastava, V. C., Singh, S., Bhatnagar, R., Mall, I. D. Electrochemical treatment of alkali decrement wastewater containing terephthalic acid using iron electrodes *J. Taiwan Inst. Chem. Eng.* **45**, 908–913 (2014).
27. Marchionni, A., Bevilacqua, M., Bianchini, C., Chen, Y.-X., Filippi, J., Fornasiero, P., Lavacchi, A., Miller, H., Wang L., Vizza,F. Electrooxidation of Ethylene Glycol and Glycerol on Pd‐(Ni‐Zn)/C Anodes in Direct Alcohol Fuel Cells. *ChemSusChem.* **6 (3)**, 518–528 (2013).
28. Hong, W., Shang, C., Wang J., Wang, E. Trimetallic PtCuCo hollow nanospheres with a dendritic shell for enhanced electrocatalytic activity toward ethylene glycol electrooxidation. *Nanoscale.* **7**, 9985–9989 (2015).
29. Garg, K. K., Prasad, B., Srivastava, V. C. Comparative study of industrial and laboratory prepared purified terephthalic acid (PTA) waste water with electro-coagulation process. *Sep. Purif. Technol.* **128**, 80–88 (2014).
30. Adschiri, T., Lee, Y.-W., Goto, M., Takami, S. Green materials synthesis with supercritical water. *Green Chem*. **13**, 1380–1390 (2011).
31. Verma, S., Prasad , B., Mishra, I. M. Thermochemical treatment (thermolysis) of petrochemical wastewater: COD removal mechanism and floc formation. Ind. *Eng. Chem. Res.* **50**, 5352–5359 (2011).
32. Kleerebezem, R., Beckers, J., Hulshoff Pol, L. W., Lettinga, G. High rate treatment of terephthalic acid production wastewater in a two‐stage anaerobic bioreactor. *Biotechnol. Bioeng.* **91**, **(2)**, 169–179 (2005).
33. Cocero, M. J., Alonso, E., Torı´o, R., Vallelado, D., Sanz, T., Fdz-Polanco, F. Supercritical water oxidation (SCWO) for poly (ethylene terephthalate)(PET) industry effluents. *Ind. Eng. Chem. Res.* **39**, 4652–4657 (2000).
34. Hu, L.-C., Oku, A., Yamada, E. Alkali-catalyzed methanolysis of polycarbonate. A study on recycling of bisphenol A and dimethyl carbonate. *Polymer.* **39**, 3841–3845 (1998).
35. Yim, K.-J., Song, D.-K., Kim, C.-S., Kim, N.-G., Okuyama, K., Ogi, T., Iwaki, T., Lee, S.-E., Kim, T.-O. Selective, high efficiency reduction of CO_2_ in a non-diaphragm-based electrochemical system at low applied voltage. *RSC. Adv*. **5**, 9278–9282 (2015).
36. Kao, C.-Y., Wan, B.-Z., Cheng, W.-H. Kinetics of hydrolytic depolymerization of melt poly (ethylene terephthalate). *Ind. Eng. Chem. Res.* **37**, 1228–1234 (1998).
37. Butkovskyi, A., Jeremiasse, A. W., Hernandez Leal, L., T. Zande, V. D., Rijnaarts, H., Zeeman, G. Electrochemical conversion of micropollutants in gray water. *Environ. Sci. Technol.* **48**, 1893−1901 (2014).
38. Ren, D., Deng, Y., Handoko, A. D., Chen, C. S., Malkhandi, S., Yeo, B. S. Selective Electrochemical Reduction of Carbon Dioxide to Ethylene and Ethanol on Copper (I) Oxide Catalysts. *ACS Catal.* **5**, 2814−2821 (2015).
39. Nakata, K., Ozaki, T., Terashima, C., Fujishima, A., Einaga, Y. High‐Yield Electrochemical Production of Formaldehyde from CO_2_ and Seawater. *Angew. Chem.* **126**, 890 –893 (2014).
40. Collins, M. J., Zeronian, S. H. The molecular weight distribution and oligomers of sodium hydroxide hydrolyzed poly (ethylene terephthalate). *J. Appl. Polym. Sci.* **45**, 797–804 (**1992**).
41. Liang, B., Long, Z. Y., Qing, H. Y., Bing, Z., Yi, P. S. Safe and convenient procedure for solvent purification. *J. Nat. Gas Chem.* **5**, 3(1996).
42. Berkes, B. B., Inzelt, G., Schuhmann, W., Bondarenko, A. S. Influence of Cs^+^ and Na^+^ on specific adsorption of *OH, *O, and *H at platinum in acidic sulfuric media. *J. Phys. Chem. C.* **116**, 10995–11003 (2012).
43. Gu, J., Yan, Y., Krizan, J. W., Gibson, Q. D., Detweiler, Z. M., Cava R. J., Bocarsly, A. B. p-Type CuRhO_2_ as a Self-Healing Photoelectrode for Water Reduction under Visible Light. *J. Am. Chem. Soc.* **136**, 830−833 (2014).
44. Ma, F., Hanna, M. A. Biodiesel production: a review. *Bioresour. Technol.* **70**, 1–15 (1999).
45. Bai, L., Zhao, Y.-L., Hu, Y.-Q., Zhong, B., Peng, S.-Y. The natural selection of the chemical elements. J. Nat. Gas Chem. **5 (3)**, 229–236 (1996).
46. Jeon, I.-Y., Choi, H.-J., Ju, M. J., Choi, I. T., Lim, K., Ko, J., Kim, H. K., Kim, J. C., Lee, J.-J., Shin, D., Jung, S.-M., Seo, J.-M., Kim, M.-J., Park, N., Dai, L., Baek, J.-B. Direct nitrogen fixation at the edges of graphene nanoplatelets as efficient electrocatalysts for energy conversion. *Sci. Rep.* **3**, 2260 (2013).
47. Qiao, J., Xu, L., Ding, L., Shi, P., Zhang, L., Baker, R., Zhang, J. Effect of KOH Concentration on the Oxygen Reduction Kinetics Catalyzed by Heat-Treated Co-Pyridine/C Electrocatalysts. Int. J. Electrochem. Sci. **8**, 1189 – 1208 (2013).
48. Wu, L., Li, S., He, W., Teng, D., Wang, K., Ye, C. Automatic Release of Silicon Nanowire Arrays with a High Integrity for Flexible Electronic Devices. *Sci. Rep.* **4**, 3940 (2014).
49. Fu, Q., Mabilat, C., Zahid, M., Brissea, A., Gautiera, L. Syngas production via high-temperature steam/CO_2_ co-electrolysis: an economic assessment. *Energy Environ. Sci.* **3**, 1382–1397 (2010).
50. Kang, P., Chen, Z., Nayak, A., Zhang, S., Meyer, T. J., Single catalyst electrocatalytic reduction of CO_2_ in water to H_2_+ CO syngas mixtures with water oxidation to O_2_. *Energy Environ. Sci.* **7**, 4007–4012 (2014).
51. Varcoe, J. R., Atanassov, P., Dekel, D. R., Herring, A. M., Hickner, M. A., Kohl, P. A., Kucernak, A. R., Mustain, W. E., Nijmeijer, K., Scott, K., Xuk, T., Zhuang, L. Anion-exchange membranes in electrochemical energy systems. *Energy Environ. Sci*. **7**, 3135–3191 (2014).
52. Page, S.-E., Arnold, W. A., McNeill, K. Terephthalate as a probe for photochemically generated hydroxyl radical. *J. Environ. Monit*. **1**2, 1658–1665 (2010)
53. Razavi, B., Abdelmelek, S. B., Song, W., O’Shea, K. E. Photochemical fate of atorvastatin (lipitor) in simulated natural waters. *J. Water Research*. **45**, 625-631 (2011)
54. Xu, H., Cooper, W. J., Jung, J., Song, W. Photosensitized degradation of amoxicillin in natural organic matter isolate solutions. *J. Water Research*. **45**, 632-638 (2011)
55. Luo, X., Zheng, Z., Greaves, J., Cooper, W. J. Trimethoprim: Kinetic and mechanistic considerations in photochemical environmental fate and AOP treatment. *J. Water Research*. **46**, 1327-1336 (2012)
56. Koper, M. T. M., Kwon, Y., van der Ham, C. J. M., Qin, Z., Koper, M. T. M. A new mechanism for the selectivity to C1 and C2 species in the electrochemical reduction of carbon dioxide on copper electrodes. *Chem. Sci*. **2**, 1902 (2011)
57. Peterson, A. A., Abild-Pedersen, F., Studt, F., Rossmeisl, J., Nørskov, J. K. How copper catalyzes the electroreduction of carbon dioxide into hydrocarbon fuels. *Energy Environ. Sci*. **3**, 1311–1315 (2010)
58. Koper, Marc. T. M., Gallent, E. P. G., Schouten, K. J. P., Structure Sensitivity of the Electrochemical Reduction of Carbon Monoxide on Copper Single Crystals. *ACS Catal*. **3**, 1292−1295 (2013).
